# Role of Coronary Artery Calcium Score CT in Risk Stratification of Asymptomatic Individuals

**DOI:** 10.3390/jcdd12110442

**Published:** 2025-11-09

**Authors:** Darío Gómez-Díaz, Pablo Díez-Villanueva, Beatriz López-Melgar, Luis Flores, Gianluca De Toffol, Agustín Ramos, Álvaro Montes, Alberto Cecconi, Jesús Jiménez-Borreguero, Fernando Alfonso

**Affiliations:** Cardiology Department, Hospital Universitario de La Princesa, Calle Diego de León, 62, 28006 Madrid, Spain

**Keywords:** coronary computed tomography, subclinical atherosclerosis, risk stratification, coronary artery calcium, cardiovascular prevention, CAC score

## Abstract

Background: The evaluation of coronary artery disease has undergone significant transformation in recent years, with increasing emphasis on the detection and characterization of subclinical atherosclerotic plaques in order to improve cardiovascular prevention. In this context, coronary computed tomography (CT) has emerged as a promising tool. Coronary artery calcium scoring (CACS) is a powerful predictor of cardiovascular events. Methods: A narrative review on the role of CACS in risk stratification of asymptomatic adults is conducted through a structured search in PubMed (2010–2025), including guidelines, consensus documents, observational studies, and clinical trials published in English. Results: CACS reliably identifies subclinical atherosclerosis and stratifies cardiovascular risk in asymptomatic individuals. Large studies, including the Multi-Ethnic Study of Atherosclerosis (MESA), demonstrate that CACS predicts 10-year cardiovascular events, improves risk reclassification beyond traditional scores, and identifies very low-risk individuals (those with a CAC of 0). Moreover, CACS retains prognostic value across age, sex, and ethnic subgroups; supports decision-making for lipid-lowering therapy and aspirin use; and correlates with non-gated CT assessments. Progression of CAC further informs risk, although its interpretation is influenced by preventive therapies and is complemented by more comprehensive plaque imaging. Conclusions: CACS is a validated tool for detecting subclinical atherosclerosis and refining cardiovascular risk in asymptomatic individuals. CACS thus complements traditional risk scores and represents a key component of contemporary and future cardiovascular prevention in clinical practice.

## 1. Introduction

Cardiovascular disease remains the leading cause of mortality worldwide, with coronary artery disease being its most prevalent manifestation [1]. An acute coronary syndrome, or even sudden cardiac death, can be the first clinical presentation in up to 50% of cases, thus highlighting the limitations of current detection strategies that solely rely on traditional cardiovascular risk factors [2].

In the last two decades, the early detection of subclinical atherosclerosis in asymptomatic individuals has emerged as a key strategy to reduce the number of cardiovascular events, since this approach may even improve primary prevention strategies, also aimed at reducing disease progression [3]. Although traditional risk scores—like the Framingham Risk Score (FRS), the SCORE-2, or the ASCVD Score—provide useful estimation of population-level risk, more than 50% of acute myocardial infarctions still occur in patients classified as “low risk” [4]. Such discrepancy has driven the focus into search for “enhancers”, which may be capable of detecting, beyond current models, hidden atherosclerotic burden and refining risk stratification in apparently healthy individuals.

Risk assessment should not be regarded as a static process, since it evolves over time not only as a consequence of aging but also due to modifications in both traditional and non-traditional cardiovascular risk factors [5]. In the specific context of asymptomatic individuals, current guidelines endorse coronary artery calcium scoring (CACS), derived from non-contrast computed tomography, as the most robust imaging-based tool for refining risk stratification [6]. In recent years, CACS has gained stronger recommendation owing to its reproducibility, prognostic accuracy, and ability to reclassify risk beyond clinical scores.

The prevalence of noncalcified plaques in low-risk patients is not negligible (estimated at around 10%), yet the event rate in this group remains very low, with rates below 1% at 2.5 years [4]. Although coronary CT angiography (CCTA) can detect subclinical atherosclerosis and characterize coronary plaque features, there is no robust evidence that its use in primary prevention improves risk classification or guides preventive therapy beyond what is achieved with CACS. In addition, CCTA involves exposure to ionizing radiation and contrast media, with associated risks and higher costs that are not justified in the absence of clinical indications or symptoms [7].

**Figure 1:**
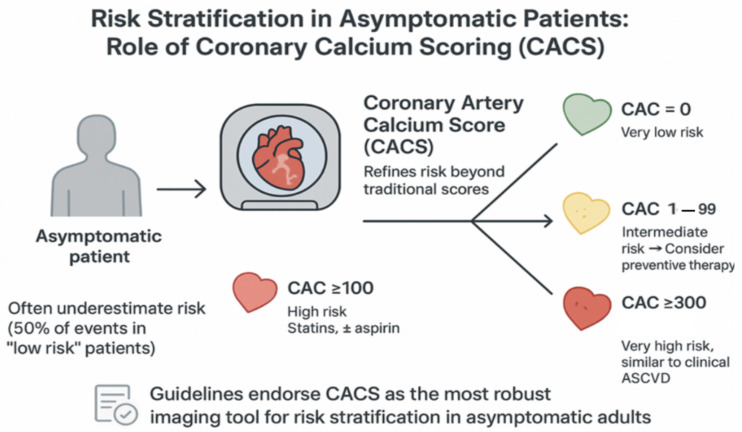
Coronary CT for subclinical atherosclerosis screening. CACS: coronary artery calcium scoring.

Hence, CACS may help to reclassify estimated risk from traditional scales and translate and integrate this information into changes in clinical practice to improve clinical outcomes [8] Figure 1.

Of note, many studies have focused on symptomatic populations, aiming to clarify the need for escalation to invasive testing or, alternatively, to optimize medical therapy for secondary prevention and then assessing long-term outcomes [9]. At the same time, there is extensive and growing evidence supporting the role of coronary CT in asymptomatic individuals with a high burden of cardiovascular risk factors or other clinical conditions, highlighting its importance in the field of primary prevention [4].

## 2. Material and Methods

This narrative review focused on the role of CACS in risk stratification of asymptomatic individuals. The clinical question was framed using a simplified PICO approach: Population—asymptomatic adults; Intervention—assessment with CACS; Comparison—traditional risk scores; Outcome—risk reclassification and prediction of cardiovascular events.

A structured literature search was performed in PubMed for articles published between 2010 and 2025. Search terms included “Coronary Artery Disease”, “Coronary Artery Disease/diagnostic imaging”, “Calcium score”, “CT”, “Computed tomography”, “Risk Stratification”, “Reclassification”, “Risk assessment”, “Low risk”, and “Intermediate risk”. Guidelines, consensus documents, observational studies, and clinical trials (retrospective, prospective, single- or multicenter) published in English and available in full text were considered.

Studies were excluded if they did not address the primary research question, included symptomatic patients, had very small sample sizes (<50 participants), or were narrative reviews. Additionally, recent AI-assisted tools, including language models, were consulted to aid in summarizing and synthesizing the literature, but all critical interpretations were independently verified and performed by the authors.

## 3. Results

### 3.1. CACS by Cardiac CT for the Screening of Subclinical Atherosclerosis

Coronary artery calcium (CAC) scoring was introduced as a surrogate marker to estimate atherosclerotic burden using CT. The Agatston score [10,11] is the most widely validated and utilized tool. It defines a calcified lesion as one consisting of at least three contiguous pixels on a 1 mm slice image with a density equal to or greater than 130 Hounsfield units [10].

Many large studies have shown that CACS can significantly reclassify patients (Table 1).

One of the most prominent studies—the Multi-Ethnic Study of Atherosclerosis (MESA) [12]—demonstrated that CACS is a robust predictor of 10-year cardiovascular events in asymptomatic individuals, independent of traditional risk factors and consistent across age, sex, and ethnic subgroups. Participants with a CAC of 0 exhibited very low event rates (<5% over 10 years), whereas those with CAC ≥ 100 experienced consistently higher risks, exceeding 7.5%, thereby establishing clinically useful thresholds for decision-making in primary prevention. Furthermore, MESA showed that incorporating CACS into risk assessment models improves both discrimination and reclassification compared with traditional risk scores, influencing international guideline recommendations for the use of CAC in asymptomatic individuals at intermediate risk.

Another classical study conducted by Erbel et al. [13], also demonstrated that in asymptomatic individuals with intermediate risk, coronary calcium quantification provided significant prognostic and reclassification value beyond traditional risk factors. Adding CACS to the Framingham Risk Score (and others) substantially increased its discriminative power, by refining classification not only for intermediate-risk individuals but also in patients at the extremes of traditional risk factor burden.

A large study by Budoff et al. [14] examined the prognostic correlation between CACS and major adverse cardiovascular events (MACE) in asymptomatic patients without prior history of disease, showing that a CACS > 300 was associated with MACE rates comparable to those with patients with established coronary artery disease, especially when CACS is equal or higher than 1,000. Furthermore, large studies such as ROBINSCA, demonstrated that participants with high CAC were more likely to seek medical advice and therefor adhere to preventive therapy than those screened with SCORE [35].

In addition, several studies have demonstrated the value of CACS when results are negative. A “zero CAC” serves as a marker identifying individuals at very low risk, with event rates of less than 1 per 1000 person-years. A study by Nasir et al. [15] in 44,052 asymptomatic individuals showed very low event rates (0.87 deaths/1000 person-years) in those with CAC = 0 compared to those with CAC > 0. Similar results were found by Le et al. [16], where MACE incidence was 2.4% in patients with CAC = 0 and 6.9% in those with CAC > 0. And the risk is even lower if the patient scores double zero in a repeated test after 5 years [17]. Another study, Valenti et al. [18] showed that CAC = 0 was associated with a vascular age substantially lower than chronological age across all age groups, and proved to be the strongest predictor of survival, outperforming traditional clinical risk scores in discrimination and reclassification. Additionally, individuals deemed high-risk by conventional scores experienced better survival if their CACS was 0 compared with low-to-intermediate risk individuals with any detectable calcium. In fact, the ROBINSCA trial demonstrated that CACS misclassified significantly fewer patients as high risk, resulting in less unnecessary preventive treatment [36]. Thus, it confirms the safety of “zero CACS” supporting delayed intervention.

Given all the above, European cardiovascular risk management guidelines recommend considering lipid-lowering therapy in patients with elevated CACS [19].

### 3.2. Role of CT in Specific Populations

Regarding sex differences, the study by Nakao et al. [20] found that women had significantly lower median CAC values than men (4 vs. 60; *p* < 0.001) and lower prevalence of ≥50% coronary stenosis, with a higher proportion of men being classified into clinically relevant risk categories (high or low) than women (58.6% vs. 24.8%). These differences reflect biological variations by sex, supporting prior evidence that on average women achieve a positive CACS 10 years later than men [21]. This may warrant sex-specific thresholds for indicating CACS. In the CAC Consortium study, the optimal age proposed to consider this test in women was above 50 years old if strong cardiovascular risk factors (CVRF) are present, or 58 years old in the absence of CVRF. Conversely, for men the optimal testing age was around 40–45 years old [22]. In contrast, when calcium is detected in the coronary arteries, the cutoff values for clinical interpretation and decision-making are the same for men and women; neither the negative predictive value of a zero CACS differs between men and women [23].

Calcium scoring also provides significant improvements in assessing cardiovascular risk in elderly asymptomatic populations (ages 55–85). The study by Elias Smale et al. [24] provided empirically derived CACS thresholds of 50 AU for reclassifying to low risk and 615 AU for high risk in intermediate-risk individuals, underscoring the value of calcium scoring for stratifying cardiovascular risk.

In high-risk patients with a family history of premature ischemic heart disease, conventional risk scores identified only 59–67% of patients who later presented with CAC > 100 AU and would therefore be eligible for statin therapy according to guidelines. Accordingly, coronary CT plays a critical role in refining risk selection in primary prevention [25].

In patients with familial hypercholesterolemia, guidelines recommend continuing lipid-lowering therapy even if CACS = 0 due to the lifelong elevated risk. However, a prospective cohort study showed that individuals with familial hypercholesterolemia and CACS = 0 still had a very low 10-year event rate (0–1.2%), suggesting that CAC may help reduce treatment costs in selected populations [26].

CACS varies significantly by race and ethnicity. In multiple U.S. cohorts, white individuals exhibit higher prevalence and burden of CACS compared to black, Hispanic, and Asian individuals, even after adjustment for traditional cardiovascular risk factors [27]. Interestingly, although black individuals generally have lower detectable CACS, their cardiovascular event risk for a given CAC score is higher, suggesting racial differences in its prognostic significance.

Current evidence indicates that CACS in athletes should be interpreted with caution, as the presence of coronary calcium reflects subclinical atherosclerosis and is associated with an increased risk of cardiovascular events, even in physically active individuals. In professional athletes, the prevalence of calcified coronary plaques is higher compared with sedentary counterparts. However, these plaques tend to be more stable and less prone to acute events, which may modify the prognostic significance of CACS in this population [27].

A CACS of 0 in athletes is associated with a low risk of significant coronary artery disease and cardiovascular events. Nevertheless, in younger athletes (<45 years), a low or null CACS does not exclude the presence of non-calcified coronary disease, which may be relevant in the context of sudden cardiac death [27].

### 3.3. Value of Coronary Calcium Detection in Non-Coronary CT

Several studies have demonstrated a correlation between CAC measured on non-gated chest CT and the Agatston score obtained from ECG-gated CT [28,29]. Although non-gated CT may overestimate calcium due to artifacts [30] or miss small lesions from thicker slices, low-dose chest CT used in lung cancer screening has shown good correlation with standard calcium scoring (concordance correlation coefficient ≥ 0.81) [30]. In addition, coronary calcium can also be visually assessed in a standardized way according to CACS categories with prognostic significance recognized in current guidelines and consensus documents [37], encouraging the inclusion of information on coronary calcification in the reports of thoracic CT.

### 3.4. Progression of Coronary Artery Calcification

Beyond its static measurement, CAC progression has been associated with increased cardiovascular events. Given the dynamic nature of cardiovascular risk factors, it is recommended to reassess risk using CACS every 5–10 years, as the “warranty period” for the development of CAC is estimated at 3–7 years [31]. Some studies have even demonstrated a nearly 15-year period of protection against mortality for individuals at low to intermediate risk, independent of age or sex [17]. Yet, one study [16] found that only 2% of individuals with CACS = 0 progressed to CACS > 50 in five years. Moreover, only those with low but nonzero initial CAC (1–100 AU) showed substantial progression (e.g., 10-year risk > 7.5%).

The ARIC study has provided compelling evidence that maintaining a favorable cardiovascular risk profile from midlife into older age significantly increases the likelihood of having a CACS of zero in later life [32]. In a 30-year follow-up of community-dwelling adults without established coronary disease, those with sustained optimal levels of total cholesterol (<160 mg/dL), systolic blood pressure (<125 mmHg), and absence of smoking had a much higher likelihood of maintaining a CACS = 0 at a median age of 80. Individuals who had never smoked were 5.7 times more likely to have a CACS = 0 compared to persistent smokers [32].

The MESA study established a standardized method to assess CAC progression, demonstrating its relationship with mortality, although its use in routine clinical practice is limited because detection of coronary calcium typically prompts initiation of statin therapy, which alters subsequent progression [33]. Current guidelines acknowledge the potential value of CAC progression in cardiovascular risk assessment [38]. However, the Progression of Early Subclinical Atherosclerosis (PESA) study has shown that CACS lags behind other imaging modalities in detecting early atherosclerotic plaque progression, highlighting the limitations of calcium-only assessment [34].

Finally, one study led by Dzaye et al. [22] modeled the optimal age to perform the first CAC assessment in young adults according to their risk profile. The analysis showed that in men with diabetes the optimal age was 36.8 years, whereas in women with diabetes it was 50.3 years. In contrast, among individuals without risk factors, the estimated ages were 42.3 years in men and 57.6 years in women. These estimates were derived using a 25% performance threshold for detecting CAC > 0.

### 3.5. Addressing Risk with CACS

The 2022 American College of Cardiology Expert Consensus Decision Pathway (ECDP) underscored the role of CAC in guiding LDL-C targets, advocating a more nuanced, risk-informed approach to lipid management. For individuals with CAC > 100 or above the 75th percentile, an LDL-C target of <70 mg/dL is recommended, with more aggressive targets (<55 mg/dL) considered for those with CAC ≥ 300 reflecting risk levels akin to clinical ASCVD [21].

In addition, the ECDP recommends considering aspirin therapy in patients with a CAC score ≥ 100 and low bleeding risk, as in this subgroup the net clinical benefit outweighs the risk of hemorrhage. Conversely, in patients with a CAC score of 0, aspirin is not indicated.

### 3.6. Future Role/Perspectives

Looking ahead, CACS in cardiovascular prevention is expected to expand beyond its current use as a binary tool for refining risk stratification. Future applications may include integration with emerging imaging techniques that assess both calcified and non-calcified plaque burden, providing a more comprehensive view of subclinical atherosclerosis. Moreover, combining CACS with circulating biomarkers, genetic risk scores, and artificial intelligence-based predictive models may further enhance individualized cardiovascular risk assessment.

Another promising direction lies in the longitudinal use of CACS, not only for initial risk reclassification but also to monitor disease progression and treatment response. Although statin therapy modifies plaque composition and complicates the interpretation of calcium progression, ongoing research aims to clarify whether dynamic changes in CACS can serve as a surrogate marker for therapeutic efficacy.

Finally, broader implementation of CACS in population-based screening and tailored prevention strategies could optimize the allocation of preventive therapies, reduce unnecessary interventions and, ultimately, improve clinical outcomes. However, large-scale randomized trials and cost-effectiveness analyses will be essential to establish its role in routine clinical practice.

## 4. Conclusions

Coronary artery calcium scoring has emerged as a cornerstone in the detection of subclinical atherosclerosis and the refinement of cardiovascular risk stratification in asymptomatic individuals. With excellent negative predictive value and the ability to reclassify intermediate-risk patients, it provides a capable and validated tool to direct preventative measures. While important limitations remain, especially in younger people and in the interpretation of progress under lipid-lowering therapy, CACS still bridges the gap between conventional risk scores and imaging-based analysis, positioning itself as a key component in the current and future landscapes of cardiovascular prevention.

## Figures and Tables

**Table 1:** Main studies addressing the impact in prognosis of CAC by Coronary CT.

Study Name	Year	Study Type	Population Included	Study Results
Ibáñez et al. [3]	2021	Narrative review of the PESA study	4184 asymptomatic middle age adults (40–54 years)	High prevalence and progression of atherosclerosis even in low risk patients by traditional scores
Kim et al. [4]	2013	Retrospective observational study	2133 asymptomatic low risk patients according to NCEP	High prevalence of atherosclerotic disease
Silverman et al. [12]	2014	Prospective cohort study	6698 asymptomatic patients from MESA study	>300 UA CACS high risk CACS = 0 UA very low risk
Erbel et al. [13]	2010	Prospective Population Study	4487 Germans, 45–75 years, without CVD	CACS improves risk stratification and reclassification.
Budoff et al. (CONFIRM) [14]	2017	Prospective Multinational Registry	4511 asymptomatic individuals without CVD, mean age 57.6 years	CACS > 300 risk is comparable to secondary prevention.
Nasir et al. [15]	2012	Prospective Cohort	44,052 asymptomatic, mean age 54 years, 34% women, without CVD	CACS predicts all-cause mortality better than traditional risk factors.
Le Viet et al. [16]	2020	Retrospective Cohort	5528 patients with no prior history of coronary artery disease	CACS = 0 is associated with an extremely low risk.
Dzaye et al. [17]	2021	Prospective cohort	3116 asymptomatic adults study MESA	Very low risk progression in CACS 0 in 5–7 years.
Valenti et al. [18]	2015	Prospective cohort	9715 asymptomatic adults	CACS = 0 warranty period up to 15 years
Matos et al. [19]	2021	Multicenter Retrospective	467 participants, 53% women, mean age 60 years	CACS is useful for risk reclassification, with no clear direct impact on therapies.
Nakao et al. [20]	2018	Multicenter Prospective, subgroup analysis by sex	991 patients between 50 and 74 years old, 46% women	CACS predicts CVD with greater strength in men.
Fernández-Friera et al. [21]	2015	Prospective Cohort	4184 asymptomatic adults (40–54 years old)	High prevalence of atherosclerotic disease in other vascular territories than coronary arteries
Dzayet al. [22]	2021	Prospective Cohort	22,346 asymptomatic adults (30–50 years old)	Optimal age for first CACS depending on the patient
Budoff et al. [23]	2018	Prospective Cohort	4184 asymptomatic adults (45–84 years old) study MESA	CACS > 100 high risk and CACS = 0 low risk
Elias-Smale et al. [24]	2010	Prospective Cohort	2028 patients ≥ 55 years (mean age 69.6), without coronary artery disease	CACS improves risk classification in the elderly.
Venkataraman et al. [25]	2020	Prospective Observational Study	1059 people between 40 and 70 years old, asymptomatic but with a family history of premature CVD	CACS improves risk stratification.
Gallo et al. [26]	2021	Prospective Cohort	1624 asymptomatic adults with hypercholesterolemia	CACS improves reclassification of cardiovascular risk in this group
Christou et al. [27]	2022	Narrative review	Asymptomatic mature athletes (over 35 years old)	Greater atherosclerotic burden and proportionally greater risk, although the plaques tend to be more calcified and stable than in sedentary individuals
Azour et al. [28]	2017	Observational transversal	222 adults undergoing routine chest computed tomography and ECG-gated for coronary calcium scoring	ungated chest CT predicts with high accuracy the ranges of the Agatston score, with a strong correlation (r = 0.811) and excellent interobserver agreement (k = 0.95);
Chiles et al. [29]	2015	Observational retrospective	1575 older adults, high-risk smokers who underwent low-dose computed tomography for lung cancer screening	The presence and quantity of coronary calcium was strongly associated with coronary and all-cause mortality in a graduated manner.
Bastarrika et at. [30]	2010	Observational	48 asymptomatic adult smokers (44 men, 4 women; mean age 59.7 years) included in a lung cancer early detection program.	Excellent concordance (CCC ≥ 0.81) and no significant differences in the estimation of the total coronary calcium score compared to ECG-gated cardiac tomography
Gopal et al. [31]	2007	Prospective cohort	710 asymptomatic adults all with CACS equal to zero on initial electron beam computed tomography	62% of individuals maintained a zero calcium score during follow-up, and only 2% developed significant progression (>50 Agatston units) during the follow-up period.
Wang et al. [32]	2025	Observational prospective cohort	2044 asymptomatic community adults free of clinical coronary artery disease from the ARIC study	Low levels of total cholesterol (<160 mg/dL), systolic blood pressure (<125 mm Hg), high HDL cholesterol (>45 mg/dL), and never having smoked are significantly associated with a higher likelihood of having a coronary calcium score equal to zero.
Budoff et al. [33]	2010	Prospective cohort	4609 asymptomatic adults	Coronary calcium score progression was significantly and independently associated with all-cause mortality.
López Melgar et al. [34]	2020	Prospective cohort	3514 asymptomatic middle-aged adults (mean age 45.7 years; 63% men) from the PESA study	The progression of multi-territorial subclinical atherosclerosis was detected in 41.5% of participants in just 3 years, being more frequent in peripheral territories assessed by ultrasound than in CACS

## Data Availability

The original contributions presented in this study are included in the article.

## References

[1] Roth G.A., Mensah G.A., Johnson C.O., Addolorato G., Ammirati E., Baddour L.M., Barengo N.C., Beaton A.Z., Benjamin E.J., Benziger C.P. (2020). Global Burden of Cardiovascular Diseases and Risk Factors, 1990-2019: Update from the GBD 2019 Study. J. Am. Coll. Cardiol..

[2] Kannel W.B., Schatzkin A. (1985). Sudden death: Lessons from subsets in population studies. J. Am. Coll. Cardiol..

[3] Ibáñez B., Fernández-Ortiz A., Fernández-Friera L., García-Lunar I., Andrés V., Fuster V. (2021). Progression of Early Subclinical Atherosclerosis (PESA) Study: JACC Focus Seminar 7/8. J. Am. Coll. Cardiol..

[4] Kim K.J., Choi S.I., Lee M.S., Kim J.A., Chun E.J., Jeon C.H. (2013). The prevalence and characteristics of coronary atherosclerosis in asymptomatic subjects classified as low risk based on traditional risk stratification algorithm: Assessment with coronary CT angiography. Heart.

[5] Neumann J.T., de Lemos J.A., Apple F.S., Leong D.P. (2025). Cardiovascular biomarkers for risk stratification in primary prevention. Eur. Heart J..

[6] Mach F., Koskinas K.C., Roeters van Lennep J.E., Tokgözoğlu L., Badimon L., Baigent C., Benn M., Binder C.J., Catapano A.L., De Backer G.G. (2025). 2025 Focused Update of the 2019 ESC/EAS Guidelines for the Management of Dyslipidaemias. Eur. Heart J..

[7] Edvardsen T., Asch F.M., Davidson B., Delgado V., DeMaria A., Dilsizian V., Gaemperli O., Garcia M.J., Kamp O., Lee D.C. (2022). Non-Invasive Imaging in Coronary Syndromes: Recommendations of the European Association of Cardiovascular Imaging and the American Society of Echocardiography, in Collaboration with the American Society of Nuclear Cardiology, Society of Cardiovascular Computed Tomography, and Society for Cardiovascular Magnetic Resonance. J. Am. Soc. Echocardiogr..

[8] Abdelrahman K.M., Chen M.Y., Dey A.K., Virmani R., Finn A.V., Khamis R.Y., Choi A.D., Min J.K., Williams M.C., Buckler A.J. (2020). Coronary computed tomography angiography from clinical uses to emerging technologies: JACC state-of-the-art review. J. Am. Coll. Cardiol..

[9] Neglia D., Liga R., Gimelli A., Podlesnikar T., Cvijić M., Pontone G., Miglioranza M.H., Guaricci A.I., Seitun S., Clemente A. (2023). Use of cardiac imaging in chronic coronary syndromes: The EURECA imaging registry. Eur. Heart J..

[10] Shreya D., Zamora D.I., Patel G.S., Grossmann I., Rodriguez K., Soni M., Joshi P.K., Patel S.C., Sange I. (2021). Coronary artery calcium score—A reliable indicator of coronary artery disease?. Cureus.

[11] Alluri K., Joshi P.H., Henry T.S., Blumenthal R.S., Nasir K., Blaha M.J. (2015). Scoring of coronary artery calcium scans: History, assumptions, current limitations, and future directions. Atherosclerosis.

[12] Silverman M.G., Blaha M.J., Krumholz H.M., Budoff M.J., Blankstein R., Sibley C.T., Agatston A., Blumenthal R.S., Nasir K. (2014). Impact of coronary artery calcium on coronary heart disease events in individuals at the extremes of traditional risk factor burden: The Multi-Ethnic Study of Atherosclerosis. Eur. Heart J..

[13] Erbel R., Möhlenkamp S., Moebus S., Schmermund A., Lehmann N., Stang A., Dragano N., Grönemeyer D., Seibel R., Kälsch H. (2010). Coronary risk stratification, discrimination, and reclassification improvement based on quantification of subclinical coronary atherosclerosis: The Heinz Nixdorf Recall Study. J. Am. Coll. Cardiol..

[14] Budoff M.J., Kinninger A., Gransar H., Achenbach S., Al-Mallah M., Bax J.J., Berman D.S., Cademartiri F., Callister T.Q., Chang H.J. (2017). When does a calcium score equate to secondary prevention? Insights from the Multinational CONFIRM Registry. JACC Cardiovasc. Imaging.

[15] Nasir K., Rubin J., Blaha M.J., Shaw L.J., Blankstein R., Rivera J.J., Khan A.N., Berman D., Raggi P., Callister T. (2012). Interplay of coronary artery calcification and traditional risk factors for the prediction of all-cause mortality in asymptomatic individuals. Circ. Cardiovasc. Imaging.

[16] Le V.T., Knight S., Min D.B., McCubrey R.O., Horne B.D., Jensen K.R., Meredith K.G., Mason S.M., Lappé D.L., Anderson J.L. (2020). Absence of coronary artery calcium during positron emission tomography stress testing in patients without known coronary artery disease identifies individuals with very low risk of cardiac events. Circ. Cardiovasc. Imaging.

[17] Dzaye O., Dardari Z.A., Cainzos-Achirica M., Blankstein R., Agatston A.S., Duebgen M., Yeboah J., Szklo M., Budoff M.J., Lima J.A. (2021). Warranty period of a calcium score of zero: Comprehensive analysis from MESA. JACC Cardiovasc. Imaging.

[18] Valenti V., Ó Hartaigh B., Heo R., Cho I., Schulman-Marcus J., Gransar H., Truong Q.A., Shaw L.J., Knapper J., Kelkar A.A. (2015). A 15-year warranty period for asymptomatic individuals without coronary artery calcium: A prospective follow-up of 9,715 individuals. J. Cardiovasc. Magn. Reson..

[19] Matos D., Ferreira A.M., de Araújo Gonçalves P., Gama F., Freitas P., Guerreiro S., Cardoso G., Tralhão A., Dores H., Abecasis J. (2021). Coronary artery calcium scoring and cardiovascular risk reclassification in patients undergoing coronary computed tomography angiography. Rev. Port. Cardiol..

[20] Nakao Y.M., Miyamoto Y., Higashi M., Noguchi T., Ohishi M., Kubota I., Tsutsui H., Kawasaki T., Furukawa Y., Yoshimura M. (2018). Sex differences in impact of coronary artery calcification to predict coronary artery disease. Heart.

[21] Fernández-Friera L., Peñalvo J.L., Fernández-Ortiz A., Ibañez B., López-Melgar B., Laclaustra M., Oliva B., Mocoroa A., Mendiguren J., de Vega V.M. (2015). Prevalence, Vascular Distribution, and Multiterritorial Extent of Subclinical Atherosclerosis in a Middle-Aged Cohort: The PESA (Progression of Early Subclinical Atherosclerosis) Study. Circulation.

[22] Dzaye O., Razavi A.C., Dardari Z.A., Shaw L.J., Berman D.S., Budoff M.J., Miedema M.D., Nasir K., Rozanski A., Rumberger J.A. (2021). Modeling the Recommended Age for Initiating Coronary Artery Calcium Testing Among At-Risk Young Adults. J. Am. Coll. Cardiol..

[23] Budoff M.J., Young R., Burke G., Carr J.J., Detrano R.C., Folsom A.R., Kronmal R., Lima J.A.C., Liu K.J., McClelland R.L. (2018). Ten-year association of coronary artery calcium with atherosclerotic cardiovascular disease (ASCVD) events: The multi-ethnic study of atherosclerosis (MESA). Eur. Heart J..

[24] Elias-Smale S.E., Vliegenthart Proença R., Koller M.T., Kavousi M., van Rooij F.J.A., Hunink M.G., Steyerberg E.W., Hofman A., Oudkerk M., Witteman J.C. (2010). Coronary calcium score improves classification of coronary heart disease risk in the elderly: The Rotterdam Study. J. Am. Coll. Cardiol..

[25] Venkataraman P., Stanton T., Liew D., Huynh Q., Nicholls S.J., Mitchell G.K., Watts G.F., Tonkin A.M., Marwick T.H. (2020). Coronary artery calcium scoring in cardiovascular risk assessment of people with family histories of early onset coronary artery disease. Med. J. Aust..

[26] Gallo A., de Isla P.L., Charriere S., Vimont A., Alonso R., Muniz-Grijalvo O., Diaz-Diaz J.L., Zambon D., Moulin P., Bruckert E. (2021). The added value of coronary calcium score in predicting cardiovascular events in familial hypercholesterolemia. JACC Cardiovasc. Imaging.

[27] Christou G.A., Deligiannis A.P., Kouidi E.J. (2022). The role of cardiac computed tomography in pre-participation screening of mature athletes. Eur. J. Sport Sci..

[28] Azour L., Kadoch M.A., Ward T.J., Eber C.D., Jacobi A.H. (2017). Estimation of cardiovascular risk on routine chest CT: Ordinal coronary artery calcium scoring as an accurate predictor of Agatston score ranges. J. Cardiovasc. Comput. Tomogr..

[29] Chiles C., Duan F., Gladish G., Ravenel J.G., Baginski S.G., Snyder B., Demello S., Desjardins S.S., Munden R.F., NLST Study Team (2015). Association of coronary artery calcification and mortality in the National Lung Screening Trial: A comparison of three scoring methods. Radiology.

[30] Bastarrika G., Alonso A., Saiz-Mendiguren R., Arias J., Cosín O. (2010). Cuantificación de la calcificación coronaria mediante tomografía computarizada torácica de baja dosis sin sincronización cardiaca. Radiología.

[31] Gopal A., Nasir K., Liu S.T., Flores F.R., Chen L., Budoff M.J. (2007). Coronary calcium progression rates with a zero initial score by electron beam tomography. Int. J. Cardiol..

[32] Wang F.M., Ballew S.H., Folsom A.R., Wagenknecht L.E., Howard C.M., Coresh J., Budoff M.J., Blaha M.J., Matsushita K. (2025). Mid- to late-life traditional cardiovascular risk factor exposure and zero coronary artery calcium: The ARIC (Atherosclerosis Risk in Communities) Study. JACC Cardiovasc. Imaging.

[33] Budoff M.J., Hokanson J.E., Nasir K., Shaw L.J., Kinney G.L., Chow D., Demoss D., Nuguri V., Nabavi V., Ratakonda R. (2011). Progression of coronary artery calcium predicts all-cause mortality. J. Cardiovasc. Comput. Tomogr..

[34] López-Melgar B., Fernández-Friera L., Oliva B., García-Ruiz J.M., Sánchez-Cabo F., Bueno H., Mendiguren J.M., Lara-Pezzi E., Andrés V., Ibáñez B. (2020). Short-term progression of multiterritorial subclinical atherosclerosis. J. Am. Coll. Cardiol..

[35] Denissen S.J.A.M., van der Aalst C.M., Vonder M., Oudkerk M., de Koning H.J. (2019). Impact of a cardiovascular disease risk screening result on preventive behaviour in asymptomatic participants of the ROBINSCA trial. Eur. J. Prev. Cardiol..

[36] Van der Aalst C.M., Denissen S.J.A.M., Vonder M., Gratama J.W.C., Adriaansen H.J., Kuijpers D., Vliegenthart R., Roeters van Lennep J.E., van der Harst P., Braam R.L. (2020). Screening for cardiovascular disease risk using traditional risk factor assessment or coronary artery calcium scoring: The ROBINSCA trial. Eur. Heart J. Cardiovasc. Imaging.

[37] Hecht H.S., Cronin P., Blaha M.J., Budoff M.J., Kazerooni E.A., Narula J., Yankelevitz D., Abbara S. (2017). 2016 SCCT/STR guidelines for coronary artery calcium scoring of noncontrast noncardiac chest CT scans: A report of the Society of Cardiovascular Computed Tomography and Society of Thoracic Radiology. J. Cardiovasc. Comput. Tomogr..

[38] Ahmadi A., Argulian E., Leipsic J., Newby D.E., Narula J. (2019). From subclinical atherosclerosis to plaque progression and acute coronary events: JACC state-of-the-art review. J. Am. Coll. Cardiol..

